# Of Digitaline; Its Physiological and Therapeutical Action

**Published:** 1849-01

**Authors:** 


*Of Digitaline ; its Physiological and Therapeutical action.* By Dr. Hervieux.—Digitaline has enjoyed this uncommon advantage, —that the experiments which it has, in various countries, been submitted to, have led to similar results. M. H. relates twelve cases in which this medicine was exhibited by M. Royer, and the results observed confirm fully the data furnished by the remarks of other physicians. Employed in doses of 1-10th, 1-5th, and even 1-4th of a grain, this medicine caused neither nausea nor repugnance; neither did it occasion borborygmi, colics, or diarrhœa. In all cases the pulse fell in a very marked manner. The average of its diminution was from 22 to 36 pulsations in one minute. The action of the medicine upon the circulation began to be appreciable after two or three hours, but attained its maximum only after one, and sometimes two weeks. With regard to the action of digitaline upon the urinary organs, M. H. observed, that although the urinary secretion was not constantly increased in quantity, in all cases vesical tenesmus was present. The patient to whom the medicine was given experienced considerable benefit from its use. The dyspnœa—congestion of the face and headache, so frequently met with in disease of the heart, were greatly alleviated; and, in two cases of pulmonary disease, considerable improvement was also obtained,—a fact attributable to the diminution of the frequency of the pulse.—*London Med. Times, from Gaz. Med.*

